# Foreign

**Published:** 1841-04-03

**Authors:** 


### FOREIGN.

*Colourless Globules in the Buffy Coat of the Blood.*—When blood is drawn from the arm, coagulation usually takes place in from five to ten minutes; the whole mass in that period becoming a soft solid. As this jellying or solidification proceeds, the surface puts on various appearances in health and disease: it may be red and homogeneous, or red with a curdled flocculent aspect, variously patched with purple or buff; sometimes there is a clear transparent fluid swimming above those portions containing the red globules, and occasionally this is superseded by an opaque dirty yellow fluid, which entirely obscures all the red portions of the blood.

When the surface of the coagulum is patched with purple or buff, the blood is said to be sizy; when the clear oily-looking fluid swims at the surface, and the red globules are subsiding entirely from it, the clot will be buffed and cupped; and when the dirty yellow opaque fluid occupies the upper portion, the buffy coating will be very dense and thick.

After standing sometime the coagulated blood separates from the sides of the vessel in which it is contained, and the serum begins to exude from the coagulum: this exudation goes on for some time, and at length the *clot* is formed, which continues to contract its dimensions for at least eighteen or twenty-four hours.

It will be convenient, practically, to make a distinction between the coagulum and the clot; the former contains the whole of the constituents of the blood, and is formed generally in the short period before specified. The *clot* is the coagulum freed from the serum, and contains only the spontaneously solidified materials with the globules.

The clot, or crassamentum, in health, is altogether red, and is usually considered as consisting of two parts : the fibrine, or coagulable lymph, which gives it solidity, and the red globules. During pregnacy, in rheumatic and other inflammatory diseases, the clot exhibits two distinct portions ; the lower red, and consisting of the same materials as in health, and the other yellow whitish, or buff-coloured, forming the inflammatory crust, and consisting of coagulable lymph free from any red globules. There can be no doubt but this inflammatory crust is an important character, having some influence or share in the cause of many diseases ; being thicker, tougher, and more completely free from red globules, in those disorders of a serious inflammatory character. It is therefore of great importance to understand its real nature.

Case I.—J. B., a man aged 60: ill with acute rheumatism; pulse from 65 to 70, feeble. V. S. to 12 oz. : blood drawn into three cups. In a quarter of an hour the first portion had a purple surface, streaked with red ; the surface of the second was entirely a dirty yellowish buff, without any visible red globules; the third was similar to the first.

On gently dipping the point of the finger on the surface of the second portion, and before the coagulation had taken place, a clear colourless drop adhered to it, which, when transferred to a piece of glass, and examined by a common lens, against the light, was found to contain an immense multitude of clear colourless globules.

Case II.—G. R., a man aged 52 : ill in bed with pleuritis. V. S. to 16 oz. In five minutes the whole surface of the blood presented an opaque, shreddy, or flocculent appearance: no red particles were visible except a few round some bubbles of air. The following morning the clot was covered by a thick, tough, buffy coat; and in the cupped centre was a jelly-like matter of looser consistence. This patient was bled again the following day : blood just the same. Before the coagulation had proceeded, and while the upper portion was yet fluid, I took up three or four tea-spoonsful quite free from any red globules, and placed them in another cup : in a short time it was coagulated; and after some hours a tea-spoonful or more of clear serum had separated, leaving a tough buffy coagulum. Hence it is obvious that the first change in inflammatory blood is its separation *into two fluid* portions; the *upper* colourless, or of a yellow buff colour; the *lower*, red. The next change is the coagulation of both these portions. After this the serum separates, part exuding from the upper *buff* portion, and part from the lower *red* portion.

Case III.—J. H., aged 45: ill with acute rheumatism. V. S. to 16 oz. The blood had all the characters of Case II. In five minutes a thin pellicle or film had formed on the surface, which, in another five minutes, became thicker and more stringy : underneath this was a clear colourless portion still fluid. On dipping the point of the finger in the surface, and transferring a colourless drop to a piece of glass, an immense multitude of separate, distinct, and colourless globules (evidently lymph; or altered blood-globules, free from any colouring matter) were seen floating in the clear liquid. In this case the clot evidently contracted its dimensions, or continued to diminish in size, for twenty-four hours.

If a small portion of fresh blood is examined by the lens, or by a low power in the microscope, the red globules are usually found congregated and entangled together in ragged confused masses ; and this may be seen even by the eye alone, the blood appearing curdled.—On the other hand, the colourless globules of the buffy coat do not appear so ; they remain much more separate and distinct; and when they do coalesce, they form strings, films, or lines.

In this case I placed several drops of this clear, coagulable liquid on the glass: in a few minutes coagulation commenced in streaks and films, all of which were evidently composed by the aggregation of the globules. After this four or five tea-spoonfuls of the same fluid were placed in a clean cup, which, by the following morning, had separated with a tough buffy clot and some clean plain serum ; the former weighed 717-5 grains; the latter 677.5 grains, or 51.5 per cent. buffy clot, and 48.5 serum. This is a large proportion of serum, and shews the strength or firmness of the coagulation by which a larger proportion of serum was squeezed out than would have been the case from a clot with the red globules. The form of the clot in buffy blood is generally contracted and concave, and of a less diameter than the lower red portions.

Case IV.—M. J., a woman, aged 38, very ill with quinsy. V. S. to 14 oz. : blood drawn into two cups. In about eight minutes there was a considerable quantity of clear liquid swimming like oil on the surface of the blood first drawn, and through this a quantity of flocculent tufts, just like curdled soap, of a dirty yellowish buff colour, could be seen, and these quite obscured the red portion of the blood.

On dipping the point of the finger into the clear liquid, it somewhat disturbed the coagulating process, and a thin gelatinous film adhered to it, together with some of the still fluid portion: both were placed on a piece of glass and examined by the lens (a Coddington) against the light, when a great number of the same colourless globules could be seen, and the coagulated film appeared to be composed entirely of these globules adhering together.—Here the clot did not attain its smallest dimensions till the end of eighteen or twenty-four hours.

On the following day I bled the patient again, when the blood displayed the same appearances. Half a tea-spoonful of the clear or colourless liquid swimming on the surface was placed on a piece of glass. On examination by the lens an immense number of the same globules were found floating in it. In about five minutes the coagulation commenced by the formation of numerous opaque streaks and films, which were evidently composed by the aggregation of the globules. In a short time the whole was jellied, and some serum separated. During this change the coagulum becomes opaque, and its composition cannot then be detected.

From these experiments it is evident that inflammatory or buffy blood has two kinds of globules; the heavier red globule sinking to the bottom, and forming the lower portion of the clot, and the colourless lighter globules swimming at the top.

Müller enters pretty minutely into the cause and appearance of the inflammatory crust, but makes no mention of any globules to be found in the fluid from which it is formed. On the contrary, he distinctly states that the fibrine of the blood is really dissolved in the liquor sanguinis, and that it does not exist in the blood in the form of globules.*

I am still pursuing my investigations on this subject: in the mean time I hope this brief communication will be the means of drawing attention to the subject—now that we are fast reverting to a "modified humoral pathology."—*Mr. Addison, in Lond. Med. Gaz.*

---

*On the Auscultatory Phenomena in externally situated Aneurisms, and on the difference between an independent and a communicated Pulsation in Tumours.* By Dr. Schuk, of Vienna.—When the stethescope is placed over an aneurismal sac, or over a diffused aneurism, one perceives a very loud uninterrupted bellows-sound increasing with each pulsation. It is the more marked the more powerful the action of the heart is, the nearer the aneurism is to the heart, the rougher the wall of the artery turned towards the blood is, and the easier the blood contained in the aneurismal cavity can be put into eddying and vibratory motions by the wave of blood passing through it. The last circumstance explains why, in enormously large aneurisms with but few layers of coagulum, as well as in those of which the sac contains scarcely any thing but layers of coagulated lymph, the bellows-sound is proportionally weak. In the first of these cases the quantity of fluid is too great for all its parts to be put into strong vibration by the current of blood; and, in the second, the quantity of fluid put in motion is small, and the layers of lymph, laid one over the other, hinder, in some measure, the conduction of the sound.

When the blowing is remarkably loud, it is observable that the note of the murmur is not equally high throughout; but that at the beginning of the diastole of the artery it appears higher, and thence decreases remarkably till the next diastole.

This murmur extends for a considerable distance both towards the heart and even somewhat further towards the distal portion of the artery, and becomes weaker as it is examined at a greater distance from the aneurism.

The causes of the murmur are, first, the friction of the current of blood on the rough walls of the aneurism, or the fibrine, or on the edges of the orifice of communication between the artery and the aneurismal pouch; second, the vibration or eddying motion of the fluid blood contained in the aneurism, excited by the current of blood passing through it.

In a varicose aneurism the auscultatory signs are heard in the most striking manner. I had, not long since, two opportunities of observing this condition; in both it resulted from unfortunate venesection at the bend of the elbow. In these cases the murmur, by its intensity, becomes a whizzing sound, and the equality of the strength, as well as of the note of the sound at the different movements of the motion of the blood, is very remarkable. These phenomena are already distinct at a period of the disease when, from its short duration, but little dilatation of the wounded vein has taken place, and they are, therefore, of essential use in facilitating and supporting the diagnosis. The loudest sound is heard at that part at which the purring can be most distinctly felt with the finger; that is, where the arterial current excites the vibrations of the venous blood. With the distance from this part, the sound gradually decreases in loudness, and at last merges into a bellows-sound, which is audible only during the diastole of the artery, and which is recognised on whatever part of the limb the stethescope is placed, as well at the shoulder-joint as at the dorsal and palmar surfaces of the hand.

The phenomena just mentioned are of themselves, and for their own sakes, interesting, and they increase the probability of the diagnosis of aneurisms of all kinds. But their practical value appears especially evident in reference to the fact, that by their means one may distinguish an independently pulsating tumour, *i. e.*, an aneurism, from one to which pulsation is only communicated. In all works on surgery we constantly meet with expressions of regret that it is in many cases so difficult to determine whether a tumour which has the symptoms of both a visible and a sensible pulsation is an aneurism, or whether the motion is only communicated to a morbid growth by the artery running under it. If the tumour can be pushed aside, and if, when its position is changed, the pulsation ceases because the artery now no longer runs under it, one may be quite sure there is no aneurism. But this cannot always be done, and, in such a case,

* Physiology, pp. 124, 128.

doubt, as to the nature of the disease, must sometimes exist in the mind even of an experienced surgeon.

If by pressing it with the finger we lessen the calibre of an artery, for instance the external iliac, to a certain extent, and sufficiently for the finger to perceive the sensation of purring, we may hear *a blowing sound at the moment of the pulsation*, along the whole course of the femoral artery down to the knee, but which, though evident and easily recognised, is yet far weaker than in an aneurism. It bears the greatest resemblance to what is called the placental murmur in pregnant women, only it is for the most part of short continuance, and limited to the moment of the arterial diastole. The phenomena that are thus artificially produced not unfrequently occur naturally in consequence of the pressure of tumours or morbid products on the arteries. If the pressure is too considerable, or too slight, no blowing is produced; in the former circumstance, because the arterial walls are brought into contact, and the circulation is prevented at that part; in the latter, because the slight pressure is not sufficient to give rise to such an obstruction of the blood as to produce a murmur of the increased friction between the blood and the arterial wall. Large thyroid glands, comprising, in some measure, the carotid, afford the most frequent opportunities of testing the truth of these observations. The blowing becomes in the same proportion weaker, and at last entirely ceases as the gland gradually diminishes in size under the use of remedies. This short consideration of the matter, therefore, warrants the following conclusion:—a tumour which pulsates synchronously with the adjacent artery may be considered to be an aneurism when the murmur heard with the stethescope is loud, uninterrupted, loudest and often highest in its note at the commencement of the diastole of the artery, and gradually from that point of time decreasing in intensity; but the pulsation is only one communicated from a subjacent artery when the murmur is either altogether absent, or, if present, is heard only at the moment of the pulsation.—*Lon. Med. Gaz., from Med. Jahrb. des Orsterreichischen Staates, Bd.* xxi. *St.* 3.

---

*Two Cases of Rupture of the Carotid Artery from Sphacelus.* In a Letter to the Editor. By Mr. C. J. Mill, Surgeon, Kirriemuir.—Two cases of rupture of the carotid artery from sphacelus, followed by fatal hæmorrhage, have happened in my practice during the last six months. Both resulted from scarlatina, which had no medical treatment during the first stage. As it is of rare occurrence, and little noticed in medical writings, I transmit you a short account of them.

*Case* I.—J. M., aged three years, stout and full. At my first visit the disease had continued about six days. The rash was very prominent over all the body; almost copper-coloured, and with vesicles interspersed here and there. The fever was very intense; the pulse full and rapid, as might be expected; for the patient had been kept as hot as possible, and stimulants given " to put out the rash," as the parents said. The throat from the first had been much affected, almost preventing deglutition and articulation. The glands in the neck were swollen and painful to the touch. I immediately applied leeches, warm fomentations, and a blister to the throat. Nauseating diaphoretics, given so as to cause vomiting occasionally, purgatives, cool temperature, tepid bathing, light diet, &c., formed the rest of the treatment. By this the patient was much relieved, and in a few days could sit up a little. But a fresh accession of fever came on, and the throat became rapidly worse. I could never get a view of the pharynx, owing to the swollen glands preventing the opening of the mouth. Leeches were again ordered, and warm poultices every second hour. Matter soon began to point over the submaxillary gland. I made a puncture, and evacuated about four ounces of healthy purulent matter. As the pulse was now weak, I ordered animal jellies and wine to support the strength. The patient again rallied, and was able to sit up a little in bed several times a day. The discharge from the opening in the neck continued, the fever declined gradually, and sleep returned, until the sixth day after opening the abscess, when an unfavourable change again happened. The pulse became quick, weak, and irregular. The patient lay on his back with a clammy cold and pale countenance, the features having that expression called hippocratic. Involuntary dejections, &c. foretold a fatal termination soon. I mentioned to the friends that the child was fast sinking. On the evening of that day a sudden and most violent discharge of arterial blood took place from the mouth and wound in the neck. In the course of three minutes, about a chopin had escaped. By the time I reached the house the child was dead. A large tumour extended from the right ear, as far as the clavicle, caused by the accumulation of blood below the fascia.

Examination of the parts was not allowed.

*Case* II.—I was called to Ann ——, aged seven years, about the tenth day of the fever, which was still intense. The throat had been and still was severely affected; but the eruption was completely gone. The pulse, though rapid, was weak. I did not consider leeching proper, but applied a blister and hot poultices. Afterwards gentle laxatives, nourishing diet, and wine, were given. The symptoms continued much the same for eight days. On the evening of the 18th day from the date of seizure I saw the child, and told that death would soon happen. In about two hours I was sent for owing to a most profuse discharge of blood from the mouth having occurred. Before I reached the house the child was dead.

No examination was allowed.

In such cases as these nothing can be done. For it is owing to the disease being neglected in its first stage, that it is so severe afterwards. The practitioner ought to bear in mind the possibility of such an occurrence in cases of neglected pharyngeal inflammation; though fortunately they are rare.

I think that the inflammation in both cases had commenced in the mucous membrane covering the pharynx, and, being aggravated by mal-treatment, had terminated in sphacelus; and that it had spread from the mucous membrane of the throat to the tonsil and cellular tissue and sheath surrounding the blood-vessels. My reason for this opinion is, that in scarlatina the mucous membrane of the throat is always more or less primarily inflamed; and in many cases the inflammatory action went on steadily increasing, until the fatal termination. The constitutional symptoms already given indicated mortification. The blood was decidedly arterial. It came so rapidly and suddenly, as clearly to indicate the perforation of a large blood-vessel. From all these reasons, therefore, I considered myself perfectly warranted in terming the cases sloughing of the artery. Dissection would have set this question completely at rest; but to that, popular prejudice in this northern quarter forms as yet an almost insuperable barrier.—*Edinburgh Medical and Surgical Journal.*

*On Ophthalmia Neonatorum.* By Dr. P. G. Cederschjöld.—In order to ascertain whether the purulent ophthalmia of new-born infants is caused by a discharge from the genitals of the mother, which affects the eyes of the child during birth, Dr. Cederschjöld had the question put to every woman delivered during the year 1832 in the general lying-in hospital at Stockholm, whether or not she had such a discharge. Three hundred and sixty women were delivered; and subtracting those who bore dead children, or whose children died a few days after birth, there remained 328. Of these, 137 had a discharge from the genitals, and 181 had not. Thirty infants had purulent ophthalmia; namely, 20, whose mothers had a discharge, and 10, whose mothers had none. Hence it appears that discharges from the genitals are extremely common among pregnant women; that women may be afflicted with them, without giving their offspring ophthalmia purulenta; and that children may have the disease, though the mothers have no discharge; a proof that the malady may arise from other causes. But when we consider that of the children born of mothers affected with a discharge, 20 in 137, or about 1 in 6 [more nearly 1 in 7] suffered from the opththalmia; while only 10 in 181, or about 1 in 18, of those whose mothers were unaffected, had the opththalmia, and that, therefore, the proportion of the former was [nearly] three times as great as of the latter, we may assume, *that a discharge from the genitals of the mother, though not the sole cause of this disease, is a very frequent one.*—*London Medical Gazette, from Zeitschrift für die gesammte Medicin, from a Swedish work.*

*On the Lymphatics of the Brain.* By F. Arnold.—In his researches on the structure of the brain and spinal cord, and in his Icones Anatomicæ, the author has published the first complete description of the absorbents of the brain. In the dura mater, veins only were for the most part filled by the injection, but he obtained very successful mercurial injections of the absorbents of the pia mater. He distinguishes three different networks: 1st, a superficial delicate one, whose vessels are one-sixth of a line in diameter, and whose meshes are extraordinarily minute, lying in the cellular tissue which connects the arachnoid with the pia mater; 2d, a deeper and coarser network also in the subserous tissue of the arachnoid, and composed of vessels with a diameter of one-fourth of a line; 3d, a still coarser network, with vessels one-half of a line in diameter, and with such narrow interspaces, that when they are filled the brain seems completely covered by the injection. This network, however, is contained in the pia mater, and passes with its plexus into the depths of the furrows between the convolutions. The trunks of the absorbents into which these networks are continued take the course of the veins on the surface of the brain, accompany them closely, and pass with them and with the arteries through the foramina in the skull. In the choroid plexuses of the lateral and third ventricles there are lymphatic networks and small trunks, which collect into one considerable trunk, accompanying the vena magna Galeni. The absorbents of the very substance of the brain, which probably go with its small veins, could not be filled; they always gave way just on the walls of the ventricles. At present also the attempts to inject the absorbents of the spinal cord have had no success.—*Med. Gaz.*

*Dissection of a Club-foot,* in which the sole was turned inwards and upwards, the patient treading on the upper surface of the os cuboides, which had become rough and irregular, giving rise to an extensive ulceration on the instep, for which the limb was amputated; but the case did not do well, owing to the formation of abscesses in the thigh, and a subsequent attack of hemiplegia.

Tendo achillis very much shortened, preventing flexion of the ankle-joint.

Tendon of the tibialis anticus very short, preventing eversion of the foot.

Tendon of the extensor longus pollicis rather shortened, but nearly natural.

Tendons of the extensor longus digitorum natural.

Tendon of the peroneus tertius elongated and expanded.

Extensor brevis digitorum flabby and very thin, but otherwise natural.

Tendon of the flexor longus pollicis rather shortened.

Tendons of the flexor longus digitorum somewhat shortened, but very slightly so.

Tendon of the tibialis posticus slightly shortened.

Tendons of the peronei natural.

Short muscles of the foot natural.

The bones of the foot, although the patient was twenty-seven years of age, and the disease congenital, were apparently in a natural state, and would certainly, judging from their appearance, have allowed the foot to have regained its natural position had the opposing tendons been divided. The principal opposing tendons were those of the gastrocnemeii and soleus, and the tibialis anticus, so that by dividing these tendons, the remainder, which were nearly in their natural state, would have permitted the foot to have righted itself. I am not aware that there is any novelty in this case; but as in new operations every fact possesses some value, I have sent this dissection for publication.—*Ibid.*

---

*Violent convulsions in a puerperal woman, which continued after delivery.*—The woman, who had been in excellent health up to the day she expected to be confined, was suddenly attacked with vomiting and spasms, which soon attained a frightful height; her face was swollen and pale; her eyes fixed and red; foam issued from her mouth; her respiration was stertorious, and the pulse quick and tense. The bowels were open. The paroxysms returned every quarter of an hour. Meanwhile, she was delivered by the forceps; the child was dead, and unusually small, but at its full time. Antispasmodic remedies could not be swallowed. Bleeding, clysters of assafœtida and laudanum, and rubbing the abdomen with camphorated oil did no good; the spasms continued after delivery. Ten leeches were applied to the temples, venesection was repeated, and morphia was administered endermically, at the pit of the stomach. Yet, though the lochia were regular, and the body open, the convulsions returned every quarter or half hour for two days. They then ceased, but were followed by wild phantasmata, which did not cease till the ninth day after delivery. For four days the woman was perfectly speechless.—*Ibid.*

---

*Daily revolutions of the Pulse.*—The doctrine of Cullen and other physiologists, that the healthy circulation is subject to a double diurnal excitement, and that the chief period of excitement occurs in the evening, was first disproved by Dr. Knox, in 1816. He found that there was only one diurnal revolution, independently of incidental excitements; that the pulse is more frequent and excitable in the morning on awaking, gradually becomes less so towards evening, and acquires its greatest state of depression about midnight or before going to sleep. The writer having, in ignorance of these investigations, made some experiments of the same nature, a few years afterwards, he can confirm the results at which Dr. Knox arrived, except that instead of observing actual excitement of the pulse in the morning, he found only very marked excitability. Under a careful avoidance of all accidental stimuli, such as food, exercise, mental excitement, and the like, he found no difference whatever either in the pulse or animal heat, in the course of the whole day and night; but on awakening in the morning, there was so great excitability, that trifling stimuli raised considerably the pulse and temperature; after mid-day this excitability gradually decreased; and towards midnight it was lower than at any previous period. It is remarkable, therefore, that the ordinary period of greatest excitement in hectic fever, continued fever, and many other febrile diseases, occurs exactly at the time when there is the least exitement or excitability in the healthy state of the functions.—*Dr. Christison, in the Library of Practical Medicine*, vol. i. p. 287.

---

*Purification of Sesquioxide of Iron, intended to be used as an antidote to Arsenic.*—It has been known for some time that metallic iron contains arsenic; and it has been asserted lately by M. Couerbe that all specimens of the oxide also contain arsenic. From a communication on this subject, published in a recent number of the Literary Gazette, it may be collected that iron, very frequently, but not always, contains arsenic; and that the latter metal exists in the best specimens of English, French, and Swedish iron. It is probable, therefore, that the sesquioxide (exammonia) frequently contains arsenic.

Dr. Schafhaenthl gives the following directions for obtaining the purified oxide.

"The salts of iron from which the hydrate of the peroxide of iron is to be prepared for medical purposes, ought to be perfectly freed from arsenic, by dropping the neutral solution of the iron salt into sulphydrate of ammonia. After digestion for a few hours in a moderate temperature, the precipitated sulphuret of iron will be perfectly free from arsenic as well as sulphuric acid, and after being washed upon a filter, may be dissolved in aqua regia, and then used for the preparation of the hydrate."

The importance of purifying the oxide to be used as an antidote to arsenic is so obvious, that it is hardly necessary to urge the propriety of attending to this precaution.

It is evident that the mere detection of arsenic in the alimentary canal, &c., cannot be deemed conclusive of poisoning, in instances in which the impure oxide has been administered, inasmuch as that substance often contains arsenic; but, nevertheless, we think that the cases in which the discovery of arsenic, by analysis, should be considered insufficient to prove that substance to have been the cause of death, may be reduced to a very small number indeed, by attention to the following very simple rules:—1st. A portion of the oxide should be preserved and analyzed, in order to ascertain the absence or presence of arsenic, and, if it be present, its relative proportion. 2d. The entire quantity of oxide administered should be noted down. 3d. The whole intestinal canal, as well as the stomach, the contents of the stomach and the matter vomited, &c., should be submitted to analysis. 4th. The arsenic procured by the medico-legal analysis should be accurately weighed. In this way data may be obtained which may enable us to conclude that the arsenic extracted by analysis *had not been introduced in union with the oxide of iron*, or, at all events, that the *whole of it could not have been thus introduced.*

Orfila's process should be adopted in such cases.—*Lon. Med. Gaz.*

---

*Arsenic a constituent of the blood.* By M. Vanden Broeck.—M. Vanden Broeck has recently addressed a long memoir to the Belgic Academy of Sciences, in which he endeavours to demonstrate that venous, and especially arterial blood, when treated with Marsh's apparatus, give arsenical spots of an unequivocal description. The following is the author's mode of experimentation, with tests, the purity of which had been previously established.

The serum of the blood is dried until decomposition begins to take place; a small quantity of nitrate of potass is then added, and heat applied, until the mass is carbonized. When cooled, this mass is washed with water, filtered, saturated with sulphuric acid, and placed in Marsh's apparatus. By this means certain spots of a doubtful character are obtained. The carbonized residuum, which remained on the filter, is now taken; some sulphuric acid, enough to form a paste of it, is added, and the whole exposed for ten or twelve hours to the air. Heat is then applied for about twenty minutes, small quantities of water being gradually added, and the mass is boiled for two hours. Small bits of pure carbonate of soda are thrown in occasionally, and when completely saturated, the mass is evaporated to dryness. It is now re-dissolved in water, so as to suspend the carbonized mass, and the solution is placed in Marsh's apparatus. In this manner the author succeeded in obtaining from venous and arterial blood as many as ten large spots of arsenic, of a brown colour, volatile and strongly glittering.—*London Provincial Medical and Surgical Journal.*

---

*Case in which six pregnancies occured during amenorrhœa.* By Dr. Flechner, of Vienna. A woman, now 35 years old, menstruated for the first time in her fourteenth year, being then but imperfectly developed. Menstruation continued regular for some time, but then ceased for nine months, during which time many symptoms of chlorosis appeared. She then fell into a feverish condition, which continued for several weeks, but left her in a better state of health than she had previously enjoyed. During her convalescence the catamenia reappeared, and they now observed a regularly periodical type for several years, though still usually accompanied by congestion of blood about the head and chest. In her twenty-second year she married, and during the next year gave birth to a healthy child, which she suckled for only a few months, for her supply of milk then decreased, though no traces of return of the process appeared. Instead of the latter came on periodically pains in the head varying in severity and length of continuance, with a sensation of pressure and heat in the frontal and parietal region, and frequently great distress of breathing and palpitation of the heart, or even more or less distinct paroxysms of asthma. Notwithstanding the physiological energy of the uterus seemed completely paralysed, yet after two years she again became pregnant. Gestation and parturition were gone through in the same manner as at first time; and then, in the place of the catamenia, which were still suppressed, the same train of periodical symptoms again came on. In this way the woman became a mother six times in the course of thirteen years, without a trace of menstrual fluid ever once appearing; and she declared that during this time she had observed neither a blennorrhœa nor any morbid secretion of the uterine or other system, that could be deemed vicarious of the catamenia.

Cases of conception during lactation, before the return of the catamenia, are by no means rarities, and the secretion of milk continued for a year sometimes hinders the secretion of menstrual blood without at all excluding the possibility of conception. But in this case the peculiar condition induced by lactation could have had nothing to do with the suppression of the menses.—*Med Gaz. from Medicinische Jahrbücher Osters Staates.*

---

*Transposition of the viscera.*—Dr. Ewertzen, of Hilleröd, reports a medico-legal dissection of a boy nine years old, in whom several viscera of the abdomen (namely, the spleen, the transverse portion of the colon, and parts of the small intestines,) were found in the thorax. He supposes they had been there from birth.—*Danish Med. Rep.*

---

*Iodurct of potash in syphilis.*—In a case of inveterate syphilis with tophi, violent pains at night, and sleeplessness, Dr. Möller used ioduret of potash with evident mitigation of the symptoms. Two drachms were dissolved in eight ounces of water, and the dose was a tablespoonful three times a day. Dr. Möller convinced himself of the effect of the remedy on the system by a chemical examination of the urine; and when the patient had taken an ounce of the salt, he was freed from his complaints, got an appetite, slept well, and was better, as he said, than he had been for many years. He had been previously treated with mercurials and other remedies, by different physicians, and is now perfectly well.—*Ibid.*

*Case of Absence of the Pulmonary Artery.* By M. Bigger.—An infant which, from the fifth day after birth, had been affected with cyanosis, died at the age of five months and a half, of convulsions and dyspnœa. The left auricle and ventricle of the heart were found to be much smaller than those of the right side. The *foramen ovale* was still patent, but was closed with a perfect valve. The pulmonary veins were much contracted, but no traces of the pulmonary arteries could be detected. The aorta arose from the septum of the two ventricles, and communicated with both, so that it received the blood from both sides of the heart. A considerable arterial branch, about the size of the *arteria innominata*, was given off from the arch of the aorta, and was distributed to the lungs.—*Edin. Med. and Surg. Journ., from Wochenschrift fur die Gesammte Heilkunde.*

*Case of Irregular Disposition of the Aorta and Pulmonary Artery.* By M. F. Ducrest.—A female child was born at the Hospital of Maternité on the 8th of June, 1840, in whom respiration was never perfectly established; the infant never uttered a single cry, and refused the breast as well as all liquids which were offered to it. The skin of the child was of a pretty deep blue colour, and, notwithstanding extreme weakness and these unfavourable appearances, it lived for ten hours after its birth.

The body of the child was fully developed; the limbs were well proportioned; and it presented in every respect the appearance of a child born at the full period.

Each lung presented a double fissure, dividing them into three lobes. When removed from the body along with the heart, and thrown into water, they floated and appeared to be healthy. The great blood-vessels, however, presented the following singular distribution. The aorta took its origin from the right ventricle, and gave off in the usual manner the two cardiac coronary arteries. The trunk of the pulmonary artery rose from the left ventricle, and, after a short course to the left of the aorta, divided into three branches, two going to the lungs, the third representing the *canalis arteriosus* uniting with the aorta towards the termination of its curvature. All these vessels were of their usual proportions. The ventricle from which the aorta originated, besides its position on the right side, exhibited all the usual characters which distinguish it from the left ventricle. The two *venæ cavæ* and the large cardiac vein terminated in the right auricle. The left auricle received the four pulmonary veins. The *foramen ovale* was furnished with a perfect valve, which opened from the right to the left auricle. The septum between the ventricles was complete. No other unusual appearance was met with in any other part of the body.—*Ibid., from Archives Generales de Medicine.*

*The late Mr. John Bell.*—This distinguished surgeon, during the eventful period of his brilliant, but chequered, career, shone as a polar star to his benighted brethren, to guide them through the perilous passes of uncertainty and doubt, and direct the rays of their feeble and flickering lamp towards fields of investigation previously unploughed save by his own adventurous hand. He was a man of unquestionable genius, of rare accomplishments, of highly embellished mind, of the most lively wit and sportive imagination, and powers of sarcasm, so keen and searching, as to wither, without effort, any medical fabric he chose to touch—a man of unwearied application, of dauntless energy, of unequalled powers of mastering any subject however abstruse and complicated, of profuse stores of general information and of boundless professional learning—a *writer* of singular merit and brilliancy, with endless copiousness of language and illustration, with fund of anecdote inexhaustible, with fondness for pleasantry inextinguishable, with matchless force of reasoning, and argumentative display irresistible, with zeal in sifting and bringing to light the most hidden treasures, with ingenuity in unravelling and explaining the most secret operations of the animal economy, in unfolding the laws that determine the effects of disease, and in framing doctrines so plausible, so lucid, so natural, so effective, so seasoned with probability, so garnished with taste, so fortified by facts and observations, as to receive the sanction and gain the support of the ablest minds—a *teacher* whose ready elocution, striking declamation, persuasive manner, clear enunciation, flexible and melodious voice, appropriate action, extensive information, powerful inductive reasoning, diversified with subtilty and wit, and amusing illustrations, never failed to secure a large and gratified audience—a *surgeon* deeply versed in the history and literature of his science, skilful in diagnosis, abounding in practical resources, wary and efficient in selection of remedies, full of enthusiasm for his patient, untiring in noting down the peculiarities, and in sketching, with his exquisite pencil, the features of each remarkable case, and, *withal*, a singularly bold, daring, dexterous, and successful operator—whose exploits with the knife were the result of profound anatomical knowledge of parts in a sound and morbid state, guided by a chivalrous spirit in facing and conquering difficulties, combined with calm deliberation and circumspect address, rarely equalled.

These remarks are not from hearsay, but from intimate acquaintance with the man, from close observation whilst enjoying his society, from seeing him act his part in public under the most trying exigencies, and from poring over, by night and by day, those vast depositories of information his "*Principles of Surgery*," volumes now laid, as it were, on the shelf, but too often consulted by writers of all countries who do not hesitate, in many instances, to appropriate, boldly, doctrines and precepts and practice, to which they can lay no shadow of a claim.

It is melancholy to think that such a man, so gifted with wonderful endowments, and powers almost superhuman, should from mere frailties, so incident to genius, and so often the effect of disease, be doomed to persecution and distress from his fellow-men, because he could not always restrain, within bounds of discretion, those sallies of wit, too often scattered, it must be admitted, with unsparing hand, around those he could not but feel to be far beneath him in talent and acquirement, but nevertheless deserving of kind consideration. Valuable as his numerous and very diversified writings proved to the world, they created for himself, at last, enemies, whose jealousy, implacable hatred, and vengeance, drove him, in disgust, from his country and his home, to perish among strangers in a foreign land. But peace to his venerated remains; "*for take him all in all, we shall not look upon his like again.*"—*Gibson's Rambles.*

---

*Case of Amputation at the Shoulder-joint, with removal of the Scapula.* By M. Renoult.—M. Renoult reported to the Academy the following interesting case, operated on by Gaetani-Bey, the celebrated Egyptian surgeon. An Egyptian workman, employed in the foundries, had his shoulder severely injured, and one of his testicles blown off, in consequence of an explosion. The humerus was shattered, as well as the scapula, and the external extremity of the clavicle. The soft parts were also severely lacerated. Amputation at the shoulder-joint was immediately performed; the fragments of the scapula extracted; and the shattered extremity of the clavicle removed, The wound was then closed as far as the injured state of the soft parts would allow, and, in spite of the serious nature of the operarion, the patient made a complete recovery, and left the hospital fifty-five days after the accident.—*Edin. Med. and Surg. Jour., from Bulletin de l'Avud. Royale de Medecine.*

---

*Delivery of Four Children at a Birth.* By M. Bourdois.—A woman who had been married for twenty-three months, was, on the seventh month of her pregnancy, delivered of a male child. Two hours afterwards a second male child was born, then a third, and after a few minutes a fourth, all of the male sex. A fresh discharge of *liquor amnii* took place before the delivery of the second child. There were, in fact, two deliveries, or a double pregnancy. One placenta had but one cord attached to it, the other had three cords. As it was necessary to introduce the hand into the uterus for their extraction, they were found to be attached to opposite sides of the uterus. The two first born and the last were apparently of equal strength, and had the look of seven months' fœtuses. The third born was much less perfectly developed, and had more the appearance of a five months' fœtus. The third born lived but a few seconds, the three others only a few hours.—*Ib., from Jour. des Conn. Med. Chir.*

---

*On the Danger of injecting Fluids into the Uterus.* By M. Hourmann.—M. Vidal recently recommended the employment of injections into the cavity of the uterus for the cure of various affections of that organ, and stated that he had found their use very efficacious, and never followed by any unpleasant symptoms. Bretonneau, Ricord, and others who had made use of this practice, had, however, arrived at a very different conclusion, as they found these injections liable to produce many disagreeable effects, acute peritoneal inflammation, and death.

M. Hourmann relates a case where violent abdominal pain, followed by metro-peritonitis, was caused by means of injecting a decoction of walnut leaves into the uterus, for the cure of an obstinate leucorrhœal discharge, which had been traced to come from the cavity of that organ. The patient recovered in consequence of a violent attack of hæmorrhage from the uterus, occurring most opportunely within forty-eight hours after the administration of the injection.

M. Hourmann, wishing to ascertain whether these dangerous symptoms could be produced from a portion of the fluid having passed through the Fallopian tubes into the cavity of the abdomen, found, on injecting fluid into the uterus after death, that such was actually the case. M. Nelaton, in repeating the same experiments, observed a still more extraordinary circumstance. The liquid injected into the uterus of a woman who had died of an attack of erysipelas of the face, did not penetrate into the Fallopian tubes, but distended one of the veins of the broad ligament of the uterus, pushing before it bells of air.—*Ib. from ib.*

---

*Case of Congenital Hernia of the Heart and Stomach.* By M. Becker.—A child was born in March, 1839, well formed, vigorous, apparently quite healthy, and was still alive when the account was published. It was affected with a hernia of the heart and stomach, which protruded from the median line beyond the thorax and abdomen. The sac in which they were enclosed was almost transparent, and allowed the included organs to be pretty distinctly seen, as well as a septum which separated the heart from the stomach. It was about three and a half inches long, by four inches broad, and was neither covered with muscles, bones, nor true cuticle, but appeared to consist of a peculiar membranous expansion. The bony and muscular parts, which usually cover and protect the heart and stomach, were wanting in this case over the median line, and had thus allowed of the protrusion of these organs.—*Ibid., from Medicinische Zeitung.*

---

*The University of Edinburgh.*—The University of Edinburgh was founded in 1582, under charter from James the Sixth, and opened for pupils in 1583. It was not, however, until 1721, that a medical department was annexed—at the suggestion of Drs. Sibbald, Pitcairne, Balfour, Burnet, and Stevenson, and by the exertions of Dr. John Monro, an eminent surgeon apothecary. The first professors were Dr. Alexander Monro, on anatomy and surgery; Dr. Sinclair on the theory of medicine; Dr. Rutherford on the practice; Dr. Alston on materia medica; and Dr. Plummer on chemistry. About the same period an attempt was made to establish a hospital, for clinical purposes, in connection with the University. It did not, however, succeed; but, upon being renewed, some years afterwards, sufficient funds were raised to enable the professors to open a small house for a few patients, and the subscriptions still increasing, a charter was granted, for an act of incorporation, by George the Second, and the foundation laid, in 1738, of the *Royal Infirmary.* From that period to the present, an intimate connection has subsisted between the institution and the University—beneficial to both. Thirty-five years after the University had been in operation, Dr. Cullen was appointed professor; soon after Dr. John Gregory, then Dr. Black and Dr. Alexander Monro, secundus; and from that period the institution became the most celebrated in Europe, and has continued to flourish beyond example.

When I commenced attendance in 1806, on lectures in the University of Edinburgh, the professors were Dr. Alexander Monro, secundus, on anatomy and surgery, in conjunction with his son Dr. Alexander Monro, tertius; Dr. James Gregory, on the practice of physic; Dr. James Home, on materia medica; Dr. Andrew Duncan, on the theory of physic; Dr. Thomas Charles Hope, on chemistry; Dr. James Hamilton, junior, on obstetrics, and Dr. Daniel Rutherford on botany. Of these the elder Monro, Drs. Gregory, Hope, and Hamilton, were the most distinguished. In June, 1809, I graduated in the University, and in August, 1839—thirty years afterwards—returned to it, and then found Drs. Monro, Gregory, Duncan, and Rutherford, no more; Dr. Monro, tertius, holding the anatomical chair; Dr. Home transferred to practical chair; Dr. Christison, elected in his place, to materia medica; Dr. Graham appointed to botany; Dr. Alison to theory of physic, and Drs. Hope and Hamilton still occupying their respective chairs. I discovered, also, that new chairs had been created—one on pathology, to which Dr. John Thomson had been appointed; another on surgery, occupied by Sir Charles Bell; a third, on military surgery, held by Sir George Ballingall; a fourth, on medical jurisprudence, of which Dr. Traill was the incumbent, and a fifth, on clinical surgery, intrusted to Mr. Syme—making in all twelve distinct chairs. The old buildings, too, erected in the time of James the Sixth, which constituted nearly one-half of the college, whilst I attended, were in 1815 pulled down, and the new, commenced upon a magnificent scale in 1789, but the greater part left unfinished, for nearly thirty years, for want of funds, was then completed, and, indeed, had been occupied by all the departments, for a considerable time.

The University is very imposing in appearance, and would, from architectural merits alone, attract attention in any part of the world. Its walls are of freestone, similar to that of most of the houses in the New Town. The eastern front, extending on South Bridge-street, two hundred and fifty-five feet, is ornamented by a beautiful portico, supported by Doric pillars more than twenty-five feet high—each formed of a singular block of stone. The western is of similar extent, and the north and south sides three hundred and fifty-eight feet in depth.—Within this extensive pile there is a large quadrangle, from which an entrance leads to the museum, library, and principal lecture rooms. Than the *Library* a finer room cannot be imagined—being a hundred and ninety-eight feet long, by fifty wide, and so lofty, as to admit of an extensive gallery, which, together with the arched ceiling, is supported by handsome pilasters. Seventy thousand volumes are contained in the room, which is, also, decorated with some fine boar-hunts by Snyders, with a Teniers, and other pictures, bequeathed to the University, I believe, by Sir James Erskine. The *Museum*, commenced by Dr. Balfour, and added to by Dr. Sibbald, in the infancy of the University, was so diminished by the decay and loss of its specimens, as to be of little value, at the time Professor Jameson took the chair of Natural History; by his unwearied zeal and industry, however, an immense collection, in almost all the departments of his branch, has been brought together; which, in conjunction with the superb and valuable specimens of ornithology, purchased in France, the mineralogical cabinet of Dr. Thompson of Naples, and very numerous zoological contributions, are sufficient to fill several large rooms and galleries, some of them nearly a hundred feet long.

The *Anatomical Museum*, formed chiefly by the three Monros, part of which still belongs to the present professor, is not remarkable for variety, extent, neatness, or beauty of the preparations, but very inferior, in every respect, to most of the private collections of London, or, indeed, of the country towns throughout England.* Nor is the Anatomical Theatre such as to reflect credit upon the college, or to correspond with the rest of the lecture-rooms, which, though small, generally, are numerous and well adapted to the purpose. Each professor, however, has, as in most other institutions, his own private collections to illustrate his lectures.—The Obstetrical has long been considered valuable, and that of Dr. Christison very extensive, and well calculated to make Materia Medica what it ought to be, always, and, for the last few years, has been with us—a *demonstrative* branch. The same may be said of the Chemical department, long rich in every variety of apparatus, and also of the Surgical, illustrated by some of the most splendid drawings and models to be found in any country.—*Gibson's Rambles.*

---

### Edinburgh Professors.

*Sir George Ballingall*, is Professor of Military Surgery in the University, to which chair he was appointed a few years since. He had, previously, served long in the army, had seen much service in India and other foreign countries, and, withal, was distinguished for close observation, extent of acquirement, peculiarly amiable disposition, and gentleman-like address. I found, from conversation with several students and young physicins, he was a favourite, had acquired reputation among them for accuracy and caution, for the substantial information he imparted, and for his clear and comprehensive way of explaining difficult subjects. He is the author of several works—one on "Fever, Dysentery, and *Liver Complaints*;" another, consisting of "*Introductory Lectures to a course of Military Surgery*;" and a third on "*Military Surgery*;" besides numerous essays in medical periodicals. Sir George is rather tall, robust, and active, of open, generous countenance, mild and amiable manners, and so quiet and unpretending, as to gain, readily, the esteem and confidence of all that form his acquaintance, and shows to great advantage in the midst of his interesting family.

*Professor Alison* is a tall, slender, fine-looking man, of very quiet manners, neat but plain in dress, with peculiarly winning, pensive, and benevolent countenance. He is son of the celebrated Episcopal divine, of Edinburgh, who gained so much reputation by his sermons and work on "*Taste.*" In every way, he appeared to me, worthy of such a father, judging from his interesting conversation, while sitting beside him at dinner. As a teacher he holds high rank, and, being quite a young man, is destined, I have no doubt, to obtain still higher eminence. His work on "*Physiology and Pathology*" is deserving of commendation.

With the reputation of Professor *Christison* most persons, in this country, are well acquainted—through his excellent "*Treatise on Poisons, in relation to Medical Jurisprudence, Physiology, and the Practice of Physic.*" I was pleased with his tall, commanding person, agreeable, sprightly conversation, artless and engaging manners; and gratified to hear from his associates, and from disinterested sources, of the high position he held in the University, as Professor of Materia Medica, Dietetics, and Pharmacy—taking great pains to illustrate his lectures, by an ample collection of preparations adapted to the purpose.

Professor *Graham*, too, as far as I could learn, was highly appreciated, not only for extensive knowledge of Botany, and zeal as a teacher of that science, but for his excellent qualities as a man. That he is highly popular with his class and the public I had reason to believe; for, being detained by engagements, and entering the room late, during the *capping*, it was easy to perceive, from demonstrations of respect afforded by the candidates and audience, that he was a welcome and well known personage. Like his associates, Drs. Alison and Christison, he is of middle age, tall, and of fine constitution; and if equanimity and good humour ever contribute to longevity, has every chance of holding, with them, his professorship for many years.

Of Professor *Traill* I saw little, either at the dinner or before. He is a short, thick, squat-looking man, with bushy, black head, and queer expression, skellies slightly out of one eye, and is very busy, bustling, and important. He had resided at Liverpool, and been transferred thence to Edinburgh, and to the chair of *Medical Jurisprudence*, which he fills, it is said, with more or less cleverness.

Mr. *Syme*, the Professor of *Clinical Surgery*, is about forty years of age, has extensive practice, and is represented as a good operator. By Sir Charles Bell, and some of his other colleagues, I found him well spoken of—both as a surgeon and a man.

*Ibid.*

---

* It is but fair, however, to say, that the preparations put up by *Dr. McKenzie*—the present demonstrator of anatomy, an intelligent and successful cultivator of his branch, who politely gave me every opportunity of examining the different departments of the University—are quite equal, in many respects, to any I saw in Europe.

